# Frequency of Helminth Eggs in Faeces of Puppies Living in Urban or Rural Environments of Mexico City

**Published:** 2018

**Authors:** Jorge-Luis DE-LA-ROSA-ARANA, Raquel TAPIA-ROMERO

**Affiliations:** 1. Laboratory of Immunoparasitology, Institute of Epidemiological Diagnosis and Reference, Ministry of Health, Mexico City, Mexico; 2. Laboratory of Parasitology, Children's Hospital of Mexico, Ministry of Health, Mexico City, Mexico

**Keywords:** Helminth, Puppy, Urban-environment, Rural-environment

## Abstract

**Background:**

The dog, *Canis familiaris*, a domestic animal that maintains close contact with humans and other animals, is considered as a potential source of zoonotic parasites. The current study aimed to determine the frequency of helminth eggs in feces of puppies of dog living in urban or rural environments of Mexico City between spring and summer of 2013.

**Methods:**

Stool samples (n=180) were analyzed by sedimentation with formalin-ether. Samples were collected from puppies living in the urban zone (n=90; stray animals) or in the rural environment (n=90; stray animals, animals with owner and animals confined to a canine control center).

**Results:**

Eggs of *Toxocara canis* (41%), *Ancylostoma caninum* (8%) and *Dipylidium caninum* (3%) were found in the rural environment but none in the urban zone. A frequency of 19% of *Toxocara* eggs was found in the canine control center, while, in stray puppies, the frequency was of 12% and 10% in animals with owner. Eggs of *Toxocara* were found in 33% samples of puppies with history of antiparasitic treatment.

**Conclusion:**

This study supports the observation of helminth population reduction in urban environments. Further studies are needed to identify the factors that affect the development and transmission of helminth eggs in urban environments.

## Introduction

In Mexico, during 2011, the estimated dog (*Canis familiaris*) population was of 18 million animals but only 5.4 million lived in a house. In 2013, it was estimated a relationship of one dog for every seven inhabitants in Mexico City (1). Since stray dogs daily produce feces, represent a risk in the transmission of zoonotic parasites. In European travelers, in the last decade, many people consult a physician after returning home from tropical areas (2). Parasitic illnesses transmitted by feral/stray small animals located in touristic places are gaining importance, but local information on frequency and prevalence of parasites sometimes is not available. Mexico is one of the most popular touristic countries, usually ranked among the top 10 countries receiving the largest number of visitors; its popularity is based on a wide variety of natural and cultural attractions, located both in urban as in rural environments. *Toxocara, Ancylostoma* and *Dipylidium* are helminths transmitted by the feces of dogs. A number of studies have been performed in Mexico City between 1997 and 2005 to determine the prevalence of helminths in stray animals confined to canine control centers located at the middle and east of the city. *Ancylostoma* frequencies varied from 23% to 67%, *Toxocara* varied between 12% and 18% while the frequency of *Dipylidium* was of 60% (3, 4). Antibodies to *Toxocara* have recorded from 56% to 67% (5, 6). Consequently, data about the prevalence of helminths in dogs were obtained from adult animals which living in urban environments, but there is no data about the prevalence of helminth eggs in puppies neither the helminth prevalence has been compared between urban and rural environments. Thus, here we compared the frequency of helminth eggs in feces of puppies living in the urban or rural environments of Mexico City.

## Materials and Methods

### Study area

This study was conducted in Mexico City, comparing two counties, the former was an urbanized area located at Northeast of the city (Miguel Hidalgo) and the second was a rural area (Milpa Alta). The urban county has great infrastructure and economic progress. The rural county is located at Southeast of the city; the stool samples were collected at the village of San Antonio Tecomitl, where the climate is sub-humid temperate with regular rainfall. Stool samples were collected from puppies (3–5 months of age) between spring and summer of 2013.

### Sample collection

After getting approval from the Ethics Committee of Institute of Epidemiological Diagnosis and Reference, Ministry of Health, Mexico, 90 samples were of stray dogs living in the urban area and another 90 were collected in the rural area.

The rural samples were grouped in stray dogs (n=30), dogs with owner (n=30) and dogs confined to a canine control center (n=30). Since the animals were not captured, no sex or weight data were taken. Age was estimated by the body size and when possible by the owner or by the veterinaries at the canine control center.

### Feces exam

Feces were examined by the formalin-ether concentration technique. Each sample (2 g) was homogenized in 10 ml of 0.9% sodium chloride. The suspension was sieved and centrifuged three times for 10 min at 500 ug, discarding the supernatant on each occasion. Button was re-suspended in 3 ml of 10% formaldehyde and left to stand for 30 sec. Then, 3 ml of ethyl ether was added and vigorously stirred for 30 sec. The suspension was centrifuged for 10 min at 500 xg. The sediment was analyzed by microscopy; firstly, at 10 x magnification and then at 40 x. Three different scrutiny of 0.050 ml with parasitological lugol were made of each sample.

## Results

**Fig. 1:**
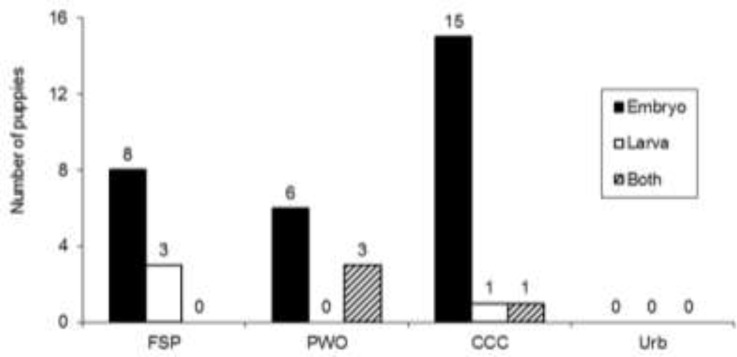
Biological development of *Toxocara* eggs. Stool samples of puppies (n=180) were analyzed by a formalin-ether technique to search for helminth eggs. Samples were obtained from urban (Urb; n=90) o rural environments. Stool samples of the rural environment were obtained from stray animals (FSP; n=30), puppies with owner (PWO; n=30) or puppies confined to a canine control center (CCC; n=30). The number of stool samples with embryonated eggs (bars closed), eggs with larvae (open bars) or a combination of embryo and larva (dashed bars) are shown

Table 1 shows the frequency of helminth eggs in feces of puppies. No parasitic structures were observed in the samples collected in the urban environment, but *Toxocara* eggs were observed in 41% of the samples collected in the rural environment, followed by *Ancylostoma* (8%) and *Dipylidium* (3%). A frequency of 19% was recorded in the canine control center, followed by the stray animals (12%) and the puppies with owner (10%). Overall, 32% of samples collected in the rural environment show embryo eggs, while in 3/90 (3%) a larva was observed inside the egg and, in another three samples a combination of larva and embryo was observed (Fig. 1). Owners of puppies were asked about the dispensation of antiparasitic drugs during the last six months before this study and, 18/30 (60%) animals received treatment with piperazine or a mixture of pyrantel and febantel; the specific treatment that each animal received could not be determined. Table 2 shows the presence of *Toxocara* eggs in 6/18 (33%) animals with treatment, while in three dogs without treatment, the eggs of *Ancylostoma* and *Toxocara* were observed simultaneously.

**Table 1:** Frequency of helminth eggs in feces of puppy dog

***Variable***		***Number of samples***	***Toxocara (%)***	***Ancylostoma (%)***	***Dipylidium (%)***
Urban environment	*Stray puppies*	90	0	0	0
	*Stray puppies*	30	11 (12.2%)	3 (3.3%)	2 (2.2%)
Rural environment	*Puppies with owner*	30	9 (10%)	3 (3.3%)	0
	*Canine control center*	30	17 (18.9%)	1 (1.1%)	1 (1.1%)
	Total Rural	90	37 (41.1%)	7 (7.8%)	3 (3.3%)

**Table 2:** Frequency of helminth eggs in puppy dogs with antecedent of antiparasitic treatment

***Variable***	***Number of puppies***	***Toxocara***	***Ancylostoma***	***No eggs***
With treatment	18	6	0	12
Without treatment	12	3^*^	3^*^	9

* These are the same puppies

## Discussion

Since Mexico City is an attractive touristic center with urban and rural zones, helminthiasis could become an important public and animal health concern. Here, we compared the frequency of helminth eggs in feces of puppies living in the urban or rural environments of Mexico City. Unexpectedly no helminth eggs were found in samples of the urban environment, while the frequency of *Toxocara* eggs in the rural area was of 41%. Eggs of *Ancylostoma* and *Dipylidium* were observed in less than 8% of the samples. The adult worms of *Ancylostoma*, *Dipylidium,* and *Toxocara* are part of the typical helminth fauna of adult dogs from Mexico City; 60% of prevalence could be usual in animals confined to canine control center (3, 4). Here, the highest frequency of *Toxocara* eggs (19%) was recorded in the canine control center followed by the samples of stray animals (12%) and animals with owner (10%). This data is important because the overcrowding allows the parasite transmission and overestimates the real prevalence of parasites. Thus, analysis of samples obtained of non-confined animals could be more realist.

In a rural area of South-eastern, Mexico (Tabasco State), *Ancylostoma caninum* was the most usual helminth, detected in 16% of examined dogs (7). However, in a feral cat population from a tropical tourist park in the same Tabasco State, 32% of *Toxocara cati* prevalence was reported between Winter 2009 and Summer 2010 (8). Thus, the season of the year has not to influence in the helminth prevalence; in contrast, the helminth biodiversity is influenced by the age of the canine. In adult dogs, the most prevalent worm was *Ancylostoma* and, *Toxocara* is the most prevalent worm in puppies, as confirmed here.

In contrast to previous studies which reported a greater number of species of helminths (3, 4), in this study, the presence of helminths was confined only to three genera (*Toxocara*, *Ancylostoma,* and *Dipylidium*), directly related with puppies. The puppies are the most welcomed by the humans and their contact with children is greater. Actually, the proximity between humans and dogs has had an increase, in addition, to being considered as a vigilant or caregiver for home or business, the dog replaces the company of humans inside the home. This phenomenon is observed in urban environments as the Mexico City; notwithstanding the dog humanization, canid habits and instincts are kept to roam streets and open spaces, which turns into a need for surveillance of parasitic infections and another zoonosis. This work is an approach to the study of this phenomenon.

Differences found between previous reports and our data may be associated with the search method to find the helminth eggs. A number of studies searched for the presence of worms by necropsy of adult dogs, while in this work, the feces of puppy dogs were analyzed by a sedimentation test, designed for eggs whose size is greater than or equal to 30–50 um in diameter. Regarding the status of biological development of *Toxocara* eggs found in 37/90 (41%) samples, a frequency of 87% was observed that enclosed embryo and 22% had larvae. Similar data were found in carrots (positivity of 1.9%) and radishes (positivity of 6.5%), where 67% of eggs were recently emitted and 33% embryonated (9). Thus, the risk of infection is greater by handling feces than consumption of vegetables. *Toxocara* eggs have been also found in parks and gardens in Mexico. In a large study in public parks, public flower beds, and home gardens in Mexico City, researchers found that 12% of the sampled sites were contaminated with *Toxocara* eggs and 90% of eggs were embryonated or larvated (10). In a study in a university campus in Mexico City, 13% of 15 gardens sampled had *Toxocara* contamination and 65% of eggs were viable (11). However, the simple exposition to the contaminated soil is not enough to become seropositive, as described in a recent study (12) to determine the association of *Toxocara* infection and gardening occupation, which shows similar risk in gardeners and controls (2%).

## Conclusion

Data here presented suggest a transition in the helminths distribution perhaps associated with the urbanization of the cities in despite the increase of the canine population in homes and on street. Further studies are needed to determine the prevalence of helminths by parasitoscopical and serological techniques between the pets and the owners. In addition, it is important to establish the factors limiting the survival of parasites in urban environments, allowing declare tourist areas as “parasite free”. We demonstrate the presence of *Toxocara*, *Ancylostoma* and *Dipylidium* eggs in the rural environment, while no helminths eggs were found in the urban area. The helminths life cycle is restricted in urban environments. Information provided by this kind of studies is essential for the implementation of strategies designed for treatment, prevention and control of these parasites.
